# Equalization of basic public services enabled by digitization: A study of mechanism and heterogeneity

**DOI:** 10.1371/journal.pone.0317207

**Published:** 2025-01-13

**Authors:** Huayi Huang, Zhibing Zhang

**Affiliations:** School of Finance, Lanzhou University of Finance and Economics, Lanzhou, PR China; IULM: Libera Universita di Lingue e Comunicazione, ITALY

## Abstract

Digitalization has penetrated into every aspect of life. However, research on the mechanisms through which digitalization affects the equalization of basic public services, as well as the heterogeneity of its impact on different fields of these services, is still insufficient. Based on the panel data of 30 provincial-level administrative regions in China from 2013 to 2023, this paper studies the mechanism and heterogeneity of the impact of digital development on the equalization of basic public services. The research finds that the improvement of the digital development level can significantly promote the equalization process of basic public services, and this finding remains robust after a series of endogeneity and robustness tests. The discussions on regional heterogeneity and spatial complexity show that digitalization makes a greater contribution to the equalization of basic public services in the central and western regions and the northeast than in the east. However, achieving equalization of basic public services across regions through digitalization is challenging. The development of digitalization and the equalization of basic public services are limited by territorial cohesion in geographical space. Underdeveloped regions have poor access to digitalization for achieving equalization of basic public services. This leads to a ‘digital divide’ between developed and underdeveloped regions. It also results in a ‘low-lying area’ phenomenon in basic public services. Additionally, the promoting effect of digitalization on the equalization of different areas of basic public services varies significantly. The effect is strongest for basic living services. It is moderate for environmental protection services and education services. The effect on medical services is not significant.

## 1. Introduction

Basic public services are the cornerstone of a country’s and society’s development. The equalization of basic public services can promote social stability and sustainable development. Continuously promoting the equalization development of basic public services is essential for people’s livelihood. In recent years, the Chinese government has highlighted the importance of promoting the equalization of basic public services. It is committed to improving the levels of education, healthcare, social security, and other basic public services for all citizens, aiming to achieve fairness, justice, and social stability. This effort has significantly narrowed the gap in basic public services between impoverished and developed regions, but some disparities still exist.

With the development and application of digital technologies, digitization has begun to permeate all industries, transforming people’s lifestyles, economic models, and social structures, thereby driving social transformation and development. In recent years, the Chinese government has actively promoted the deep integration of digital development with basic public services. The “14th Five-Year Plan for National Economic and Social Development of the People’s Republic of China and the Long-Range Objectives Through the Year 2035,” formulated in 2021, proposed that “efforts should be focused on key areas such as education, healthcare, elderly care, childcare, employment, culture and sports, and disability assistance, to promote the inclusive application of digital services and continuously enhance the sense of gain among the people.” Leveraging digitalization to promote the equalization of basic public services has become an important policy strategy of the Chinese government.

However, there are significant differences in basic public services among different groups, between urban and rural areas, and among different regions [1]. Currently, China’s public service provision is still inefficient. There are significant differences in public service demand across geographical areas, categories, and supply-demand dynamics [2]. Various categories of public services exhibit variations in terms of temporal and spatial characteristics, which consequently results in diverse effects on local economic systems [3]. Taking the Yangtze River Delta region with better economic development as an example, basic public services show a gradient decrease in the east-west direction and an inverted U-shaped distribution in the north-south direction [4]. The equalization development of basic public services is influenced by multiple factors. Digitalization is one of the fundamental element.

The process of digitalization has enhanced the beneficial influence of public services on human development [5]. Providing digital goods to vulnerable groups can bridge the service gap and enhance the ability of socially disadvantaged groups to access services [6]. Taking public healthcare as an example, the construction of digital infrastructure helps narrow the gap in access to basic public health services for China’s floating population [7]. It can increase the utilization rate of medical services, significantly improve the health levels of young and middle-aged residents and those with education levels below university, and reduce health inequality among individuals [8].

Currently, there is limited research on the empowerment of basic public services through digitalization, and most studies focus on the impact of specific aspects of basic public services, such as public health and transportation. There is a lack of comprehensive portrayal of the role of digitalization in empowering basic public services, as well as an exploration of how digitalization affects the equalization of basic public services, especially in the face of the digital divide. Questions remain about what impact digital development will have on the equalization of basic public services, whether this impact varies across different regions and sectors of basic public services, and these issues require serious study.

This paper focuses on the relationship between digitalization and the equalization of basic public services, as well as the impact of digitalization on different regions and sectors of basic public services. It explores the mechanisms by which digitalization influences basic public services and conducts empirical tests based on panel data from 30 provinces in China from 2013 to 2023. The marginal contributions of this paper are: First, it reveals the mechanisms by which digital development impacts the equalization of basic public services and validates these mechanisms empirically, providing a theoretical basis for leveraging digitalization to promote the equalization of basic public services. Second, it discusses the spatial complexity and regional heterogeneity of the effects of digitalization on the equalization of basic public services, explaining the reasons for differences in the impact of digitalization on the equalization of basic public services across different regions and the challenges of achieving equalized public services across regions using digital means. Third, it examines the heterogeneity in the mechanisms by which digitalization impacts different sectors of basic public services, laying the groundwork for accurately understanding the extent and effectiveness of digitalization’s impact on different sectors.

The remainder of this paper is organized as follows: Section 2 presents the theoretical analysis; Section 3 describes the research design; Section 4 showcases the empirical test results and further analysis; Section 5 summarizes the research conclusions and policy recommendations; and the final section outlines the limitations of this study and future directions for research.

## 2. Theoretical analysis

Basic public services are public services that ensure the basic needs for survival and development of all people and are commensurate with the level of economic and social development. The equalization of basic public services refers to the fair and accessible provision of roughly equal basic public services to all citizens, essentially improving citizens’ access to basic public services. Digitalization involves the widespread application of cutting-edge digital technologies such as the internet, big data, cloud computing, and artificial intelligence across various sectors of the economy and society, empowering comprehensive social development. It provides solutions to issues such as uneven resource distribution and information asymmetry under traditional service models.

First, digital technology can alleviate the problem of information asymmetry [9], promoting the convenient, widespread, and efficient delivery of basic public services while narrowing the gaps between urban and rural areas and among different regions, thereby enhancing the level of equalization. With the advent of the digital age, digitalization has gradually become a core driver of digital economic development, particularly in promoting the widespread and equitable supply of basic public services across various sectors, playing a significant role.

a) The application of digital technology breaks down geographical and temporal barriers, expanding the coverage of basic public services and making their supply more widespread. Through the application of digital technology, basic public services can better reach remote rural areas, addressing the bottlenecks in traditional public service delivery between developed and rural regions. For example, digital technology can improve the accessibility of health rehabilitation services [10] and deliver them to rural areas through tele-diagnosis and telemedicine, ensuring that rural residents also have access to high-quality medical care. Additionally, educational resources can be widely applied through online education, such as the digital remote education platform and educational products launched by Yunqi Company, which facilitate cross-regional, cross-school, and cross-classroom learning interactions, compensating for the shortcomings of traditional educational supply models.

b) The development of digitalization brings services closer to their audience, making basic public services more convenient. Services such as online appointment booking for medical consultations, online inquiries for public transportation information, and online applications for documents have improved the convenience of basic public services. The development of online payment platforms also allows residents to complete various living expenses payments without leaving their homes, significantly enhancing the accessibility and penetration rate of basic public services.

c) Digital technology reduces information asymmetry, promoting more efficient delivery of basic public services. Digitalization not only presents information about basic public services in electronic form but also standardizes, automates, and digitizes the processes involved in delivering these services. This avoids cumbersome manual operations and the transmission of paper documents, greatly simplifying service procedures and improving administrative efficiency.

Second, digitalization can promote the intelligence, transparency, and precision of basic public services, advancing the modernization of governance capabilities in this sector, which in turn promotes the equalization of basic public services. Government digitalization can significantly promote the equalization of basic public services between urban and rural areas [11], especially in terms of digital governance, which can effectively improve the efficiency of public services [12]. By leveraging digital technology, the government has achieved the online and mobile delivery of public service information and services. The application of e-government technology has increased public attention, narrowed the fairness gap in service provision [13], and broken down barriers between different departments and levels of basic public services, enabling resource sharing and interoperability. This has fostered cross-departmental and cross-level collaboration and exchange, allowing the public to access the services and information they need anytime and anywhere, further enhancing the efficiency of service providers. Moreover, digitalization can provide more transparent and traceable basic public services. Through digital technology, it is possible to achieve comprehensive monitoring and data analysis of the entire process of basic public services, thereby improving management and supervision effectiveness. Digitalization offers more opportunities for social participation, enabling the public to engage more widely in the decision-making and oversight of basic public services. Through online platforms and social media, the public can express their opinions and suggestions, interact with the government and service providers, thus enhancing the transparency and quality of basic public services. Additionally, digitalization can enhance the precision of basic public service provision, making resource allocation more rational. Digital platforms can provide statistical and analytical functions, delivering more precise and comprehensive data and analysis. This helps the government better understand the needs and behavior patterns of the public regarding basic public services, enabling targeted policy formulation and optimization of service provision.

Third, digitalization continuously innovates the methods of equalizing the supply of basic public services. As digital technologies are increasingly applied in various fields of basic public services, innovations and developments are constantly emerging in the infrastructure, access channels, and service providers of basic public services.

a) Digital public services based on metaverse platforms can effectively provide guidance services related to careers, entrepreneurship, and learning for enterprises and citizens [14]. The development of digitalization has empowered the innovative development of basic public service infrastructure. Intelligent infrastructure can interact with people and the environment through sensors and Internet of Things (IoT) technology, achieving automated and intelligent operations. For example, smart grids can monitor and manage electricity supply and demand through digital technology, enabling efficient and sustainable energy use. Intelligent city management systems can achieve functions such as smart traffic, smart environmental protection, and smart security through digital technology, enhancing the quality and sustainability of cities. Additionally, digital technology has significantly improved the efficiency and fairness of local government environmental governance [15].

b) Digital development has made the supply of basic public services more diverse. Traditional basic public service products were relatively single and could not meet the diverse needs of different residents. However, the empowerment of digitalization enables service providers to offer a variety of service products for the public to choose from. This not only promotes the transition of basic public service acquisition methods from traditional single channels to multiple channels but also greatly enhances the quality and efficiency of basic public service supply, meeting the diverse needs of the public.

c) The development of digitalization can drive innovation in the providers of basic public services, promoting coordinated development among multiple service providers. Basic public services are typically provided by the government or specific institutions. The emergence of digital technology not only provides a clearer understanding of market demands for basic public services to the government and specific departments but also enables the sharing of heterogeneity in service provision through digital platforms. This helps different departments target their services more precisely and provide differentiated services, achieving coordination among service providers.

## 3. Research design

### 3.1 Model selection and construction

First, to identify the impact of the level of digital development on the equalization of basic public services, this paper constructs the following ordinary panel regression model:

$$Equal_{it}=\alpha_{0}+\alpha_{1}Digital_{it}+\alpha_{i}Control_{it}+\mu_{i}+\gamma_{t}+\varepsilon_{it}$$ (1)

$$Bls_{it}=\beta_{0}+\beta_{1}Digital_{it}+\beta_{i}Control_{it}+\mu_{i}+\gamma_{t}+\varepsilon_{it}$$ (2)

$$Edu_{it}=\delta_{0}+\delta_{1}Digital_{it}+\delta_{i}Control_{it}+\mu_{i}+\gamma_{t}+\varepsilon_{it}$$ (3)

$$Ms_{it}=\lambda_{0}+\lambda_{1}Digital_{it}+\lambda_{i}Control_{it}+\mu_{i}+\gamma_{t}+\varepsilon_{it}$$ (4)

$$Es_{it}=\eta_{0}+\eta_{1}Digital_{it}+\eta_{i}Control_{it}+\mu_{i}+\gamma_{t}+\varepsilon_{it}$$ (5)

In the model, *i* represents the region, *t* represents the year, and *Equal*_*it*_ denotes the level of equalization of basic public services. Currently, there is no unified standard for the construction of the indicator system for the equalization of basic public services and the measurement of its development level. This paper refers to relevant literature [4, 16, 17] and selects four domains—basic living services, educational services, medical and health services, and environmental protection services—to construct the development level of the equalization of basic public services. These are represented by *Bls*_*it*_, *Edu*_*it*_, *Ms*_*it*_, and *Es*_*it*_ respectively, which denote the levels of basic living services, educational services, medical and health services, and environmental protection services. *Digital*_*it*_ represents the level of digital development, and *Control*_*it*_ represents control variables. *μ*_*i*_ is the provincial fixed effect, *γt* is the time fixed effect, and *ϵ*_*it*_ is the residual term. The core explanatory variable is the level of digital development, and the estimated coefficient *α*1 represents the impact of digital development on the equalization of basic public services. If the estimated coefficients *α*_1_, *β*_1_, *δ*_1,_ *λ*_1_, and *η*_1_ are significantly greater than 0, it indicates that digitalization can effectively promote the equalization of basic public services.

Second, to test the robustness of the benchmark regression and further demonstrate the marginal effects of digitalization on the equalization of basic public services at different quantiles, this paper establishes the following panel quantile regression model:

$$R\tau (Equal_{it}|Digital_{it})=\psi_{\tau 0}+\psi_{\tau 1}Digital_{it}+\psi_{\tau i}Control_{it}+\mu_{i}+\gamma_{t}+\varepsilon_{it}$$ (6)

Where *Rτ* (*Equal*_*it*_∣*Digital*_*it*_) represents the value of the equalization of basic public services *Equal*_*it*_ at the *τ*th quantile given the level of digital development *Digital*_*it*_, and *ψ*_*τ*1_ is the regression coefficient of digital empowerment at the *τ*th quantile.

### 3.2 Variable selection and description

#### 3.2.1 Explained variable: Equal access to basic public services (Equalit)

Based on the availability and consistency of data, combined with people’s basic demand for public services, a comprehensive evaluation index system consisting of 16 three-level indicators was finally constructed (see Table 1). Since the measurement methods in the existing literature are not uniform, in order to avoid the randomness of weighting and the information superposition among indicator variables, this paper adopts the entropy method in the objective weighting method to calculate the comprehensive index of basic public services to replace the equalization degree of basic public services.

**Table 1:** Index system of the development level of equalization of basic public services.

Level1 indicators	Level2 indicators	Level3 indicators	Indicator meaning	unit	weight
**Equal access to basic public services**	Basic life service	Popularization rate of water use	---	%	0.0146
		Popularization rate of gas	---	%	0.0136
		Public transport vehicles per 10,000 people	Number of public transportation vehicles (in standard units) divided by total population (10,000)	standard unit	0.0694
		Number of postal service outlets per capita	Postal business outlets (in places) /total population (10,000)	Place/ten thousand people	0.0666
	Education service	Book collection per capita in public libraries	Total number of books in public libraries /permanent resident population at the end of the year	books/person	0.1245
		Teacher-student ratio in primary schools	Number of full-time teachers in primary schools (person)/ number of primary school students (hundred people)	person/hundred people	0.0660
		Teacher-student ratio in secondary schools	Number of full-time teachers in secondary schools (person)/number of secondary school students (hundred people)	person/hundred people	0.0410
		Primary schools per 10,000 people	Number of primary schools (schools) / permanent resident population (10,000)	schools	0.0861
	Medical and health services	Number of health institutions per10,000 people	Health institutions/the permanent population at the end of the year	pieces	0.0493
		Number of beds per 10,000 people	Number of hospital beds / the permanent population at the end of the year	beds per 10,000	0.0433
		Number of physicians per 10,000	Number of practicing (assistant) physicians / the permanent population at the end of the year	persons per 10,000	0.0648
		Per capita local fiscal expenditure on health care	Local fiscal expenditure on health care / the permanent population at the end of the year	yuan	0.0937
	Environmental protection services	Green coverage rate of the built-up area	(Green coverage area of built-up area / built-up area of municipal district)* 100%	%	0.0231
		Harmless treatment rate of domestic waste	(Quantity of urban garbage treated / total amount of urban household garbage produced)*100%	%	0.0051
		Daily treatment capacity of urban sewage	---	Ten thousand cubic meters	0.1278
		Per capita urban green space area	Urban green space area / total population	hectares per 10,000	0.1110

#### 3.2.2 Core explanatory variables: Digital development level (Digitalit)

Current academic research on digitalization indicator systems and measurements is closely linked to studies on the digital economy. While there is a wealth of literature on measuring the level of the digital economy, relatively fewer studies focus solely on measuring the level of digital development, and the methods used vary. In this study, Zhang et al. (2021) and Tang et al. (2023) provide a complete assessment system for measuring the level of digital progress [18, 19]. They use digital infrastructure and digital application capabilities as two secondary indices in their evaluation. Four tertiary indexes are chosen to assess the level of digital infrastructure, based on the data availability. Additionally, six tertiary indices are picked to measure the digital application capacity, as shown in Table 2. The entropy approach is employed to ascertain the weight of each indicator, and the comprehensive index is computed to substitute the measure of digital development.

**Table 2:** Comprehensive evaluation system of digital development level.

Level 1 indicators	Level 2 indicators	Level 3 indicators	attribute	weight
**Digital development level**	Digital infrastructure	Length of long-distance optical cable line (kilometers)	+	0.0552
		Number of domain names (ten thousand)	+	0.1836
		Number of web pages (ten thousand)	+	0.3246
		Number of Internet broadband port accesses (ten thousand)	+	0.0782
	Digital application capabilities	Number of computers used per hundred employees in enterprises (sets)	+	0.0554
		Number of websites owned by one hundred of enterprises (pieces)	+	0.0163
		Proportion of enterprises with e-commerce activities (%)	+	0.0373
		Penetration rate of mobile phones (sets per hundred people)	+	0.0359
		Telecommunication business volume (RMB 100 million yuan)	+	0.1769
		Internet penetration rate (%)	+	0.0366

#### 3.2.3 Controlled variable

The equalization of basic public services is influenced by multiple factors. Referring to existing research, this paper selects economic development level (Ed), degree of government intervention (Gov), urbanization level (Urban), and industrial structure (Str) as control variables. Economic Development Level (Ed) is measured by per capita GDP in each province. To some extent, the level of economic development can reflect the development level of basic public services. Degree of Government Intervention (Gov) is represented by per capita government fiscal expenditure, which is the ratio of local general budget expenditures to the total population. Government intervention can ensure and improve people’s livelihoods, adjust regional development disparities, and to some extent, promote the provision of basic public services to more people, thereby enhancing the level of equalization. Urbanization Level (Urban) is measured by the proportion of urban population to the total population at the end of the year. As urbanization accelerates, it increases the demand for basic public services among the populace, further promoting improvements in the quality and efficiency of public service supply, which is conducive to achieving equalization. Industrial Structure (Str) is reflected by the ratio of the added value of the secondary industry to that of the tertiary industry.

### 3.3 Data sources and descriptive statistics

#### 3.3.1 Sources of date

Since 2013 is considered to be the first year of big data, some data of digital applications are also collected from 2013. In this paper, annual panel data of 30 provinces in China (excluding Tibet and Hong Kong, Macao and Taiwan) from 2013 to 2023 are selected for analysis. The data mainly come from the official website of the National Bureau of Statistics, the China Statistical Yearbook, the China Economic and Social Big Data Research Platform, and the statistical yearbook of provinces (municipalities and autonomous regions).A very small number of missing values have been supplemented by interpolation method.

#### 3.3.2 Data handling

(1) Construct the original indicator matrix. In this paper, there are 30 provinces, 16 basic public service evaluation indicators and 10 digital development indicators, forming the basic public service matrix *F* = (*f*_*ij*_) and the digital development indicator matrix *X* = (*x*_*ij*_), where *f*_*ij*_ and *x*_*ij*_ respectively represent the values of the J-index basic public service and digital development of the i province.

(2) In order to eliminate the difference of measurement units, the index is processed without dimension. The following index formula is used for processing:

$$Forward\;indicator:\;f_{ij}^{s}=\frac{f_{ij}-min(f_{j})}{max(f_{j})-min(f_{j})}$$ (7)

$$Negative\;indicator:\;f_{ij}^{s}=\frac{max(f_{j})-f_{ij}}{max(f_{j})-min(f_{j})}$$ (8)

$f_{ij}^{s}$ is expressed as the non-dimensional processing of the j basic public service indicator of the i province, that is, the standardized value.

(3) Calculate the proportion of item *j* index in which the province is *i*:

$$P_{ij}=\frac{f_{ij}^{s}}{\sum_{i=1}^{330}f_{ij}^{s}}$$ (9)

(4) Calculate the entropy value of item *j* index:

$$l_{j}=\sum_{i=1}^{330}P_{ij}*lnP_{ij},\;e_{j}=-\frac{l_{j}}{ln(330)}$$ (10)

(5) Calculate the difference coefficient of the *j*-item index:

$$d_{j}=1-e_{j}$$ (11)

(6) Calculate the weight of the *j* index:

$$w_{j}=\frac{d_{j}}{\sum_{j=1}^{16}d_{j}}$$ (12)

(7) Weighted calculate the comprehensive index of basic public service development in each province:

$$F_{i}=\sum_{j=1}^{16}w_{j}*f_{ij}^{s}$$ (13)

The level of equality is replaced by the comprehensive index of the development of basic public services, and the calculation of the level of digital development is the same as the above. Due to space constraints, the measured comprehensive index of basic public service development and digital development index of each province are not presented in this paper for the time being. Please contact the author if necessary.

#### 3.3.3 Descriptive statistics of the variables

The descriptive statistics for the main variables are shown in Table 3. From Table 3, it can be observed that the mean value of the equalization level of basic public services is 0.33052, with a maximum value of 0.58493 and a minimum value of 0.16317. The significant difference between the maximum and minimum values indicates that the current level of equalization of basic public services in China is relatively low, and there are substantial disparities in development across regions, highlighting the prominent issue of inequality.

**Table 3:** Descriptive statistical results for the primary variables.

variables	variable symbol	Observation	Mean	Std.Dev.	Min	Max
**Explained variable**	*Equal*_*it*_	330	0.33052	0.08309	0.16317	0.58493
	*Bls*_*it*_	330	0.42007	0.13908	0.03899	0.836832
	*Edu*_*it*_	330	0.30367	0.09642	0.15266	0.55969
	*Ms*_*it*_	330	0.3483	0.13957	0.05634	0.82264
	*Es*_*it*_	330	0.28568	0.15036	0.01951	0.81226
**Core explanatory variables**	*Digital*_*it*_	330	0.19977	0.12791	0.04754	0.71437
**Controlled variable**	*Ed*	330	6.50531	3.26407	2.2089	20.0278
	*Gov*	330	1.51183	0.65195	0.23214	3.87485
	*Urban*	330	61.88189	11.30128	37.89	89.6
	*str*	330	1.45251	0.93697	0.65576	11.6601

## 4. Analysis and inspection of the empirical results

### 4.1 Benchmark regression

Based on the results of the Hausman test, this paper selects the two-way fixed effects model for regression analysis, and the results are shown in Table 4. Models (1) and (2) show that, without considering control variables, whether time and region are not fixed or a two-way fixed effect is used, the results consistently indicate that an increase in the level of digital development significantly promotes the equalization of basic public services. Specifically, under the two-way fixed effect, one-unit increase in the level of digital development leads to 0.094 unit increase in the equalization level of basic public services.

**Table 4:** Regression results of the influence of digital development level on equalization of basic public services.

variables	(1)	(2)	(3)	(4)	(5)	(6)
**Digital**_**it**_	0.596*** (15.606)	0.094*** (3.208)	0.110*** (3.495)	0.111*** (3.584)	0.112*** (3.619)	0.113*** (3.631)
**Ed**			-0.031 (-1.379)	-0.059** (-2.456)	-0.075*** (-2.651)	-0.075*** (-2.654)
**Gov**				0.064*** (3.016)	0.063*** (2.953)	0.063*** (2.954)
**Urban**					-0.040 (-1.078)	-0.041 (-1.106)
**str**						-0.005 (-0.358)
**Constant**	0.192*** (30.313)	0.224*** (62.456)	0.227*** (56.719)	0.216*** (40.284)	0.232*** (14.813)	0.232*** (14.743)
**Year FE**	No	Yes	Yes	Yes	Yes	Yes
**Provincial FE**	No	Yes	Yes	Yes	Yes	Yes
**N**	330	330	330	330	330	330
**R** ^**2**^	0.449	0.908	0.909	0.912	0.912	0.912

Note: *, **, *** means the coefficients are significant at the levels of 0.1,0.05,0.01, respectively, with parentheses are the corresponding t-value.

Models (3), (4), (5), and (6) sequentially introduce control variables under the two-way fixed effect. The results show that digital development continues to significantly enhance the equalization of basic public services at the 1% significance level, and the promoting effect of digital development gradually strengthens as more control variables are added. Additionally, after introducing control variables, the R^2^ value increases compared to models without control variables, indicating an improvement in the feasibility and persuasiveness of the models, and also validating the reasonableness of the selected indicators to some extent.

From the perspective of the core explanatory variable, its significance and impact remain relatively stable as control variables are introduced. The acceleration of the digital application process, on one hand, facilitates the use of digital technology in the field of basic public services, leading to improvements in service efficiency and quality, and promoting inclusive and equitable development. On the other hand, the widespread improvement of digital infrastructure helps expand the coverage of basic public services, allowing more marginalized and impoverished areas to enjoy the same services as developed regions. Therefore, an increase in the level of digital development significantly contributes to the advancement of the equalization of basic public services.

### 4.2 Robustness test

To ensure the validity of the regression results, this paper conducts robustness checks from the following aspects:①Replacing the Explanatory Variable. Following the methodology of Bi (2024), the Digital Inclusive Finance Index (DIFI) is used to measure the level of digital development [20]. ②Changing the Measurement Method. In the previous analysis, the entropy method, an objective weighting method, was used to measure the equalization of basic public services and the level of digital development. To ensure robustness, the principal component analysis (PCA) method is employed to re-determine the weights of digital development and construct a new comprehensive index of digital development, thereby eliminating the correlated impacts among evaluation indicators. ③Winsorizing the Data. To reduce errors and biases in model regression and minimize the influence of extreme values on the regression results, the dependent variable and the core explanatory variable are winsorized at the 1% and 99% levels, and the model is re-estimated. ④Panel Quantile Regression for Robustness Testing. Regression analysis is conducted at five representative quantiles: 10%, 25%, 50%, 75%, and 90%. All test results are presented in Table 5. The results show that in various robustness check models, the level of digital development consistently and significantly promotes the equalization of basic public services, with the significance and impact magnitude similar to the benchmark regression results, indicating that the empirical results of this paper are robust and valid.

**Table 5:** Results of the robustness test.

variables	DIFI	PCA	Winsorize	τ = 0.1	τ = 0.25	τ = 0.5	τ = 0.75	τ = 0.9
**Digitalit**	0.178*** (3.850)	0.117** (2.555)	0.135*** (4.294)	0.081*** (3.741)	0.089*** (2.706)	0.113** (2.207)	0.164*** (5.694)	0.203*** (9.102)
**Ed**	-0.123*** (-3.634)	-2.186*** (-8.046)	-0.139*** (-4.223)	-0.030 (-1.507)	-0.020 (-0.669)	-0.011 (-0.226)	-0.041 (-1.563)	-0.095*** (-4.664)
**Gov**	0.076*** (3.515)	0.950*** (4.573)	0.094*** (3.796)	0.038** (2.532)	0.052** (2.313)	0.080** (2.266)	0.057*** (2.899)	0.064*** (4.179)
**Urban**	-0.063* (-1.668)	0.443 (1.223)	-0.008 (-0.195)	0.031 (1.218)	0.037 (0.953)	0.044 (0.714)	-0.035 (-1.012)	-0.052* (-1.958)
**Str**	-0.005 (-0.318)	-0.016 (-0.113)	-0.008 (-0.476)	0.003 (0.331)	-0.001 (-0.057)	-0.003 (-0.122)	-0.004 (-0.271)	-0.010 (-0.894)
**Constant**	0.234*** (14.954)	0.932*** (6.122)	0.244*** (13.334)	0.271*** (8.824)	0.261*** (5.600)	0.244*** (3.340)	0.353*** (8.633)	0.381*** (12.009)
**Year FE**	Yes	Yes	Yes	Yes	Yes	Yes	Yes	Yes
**Provincial FE**	Yes	Yes	Yes	Yes	Yes	Yes	Yes	Yes
**N**	330	330	330	330	330	330	330	330
**R2**	0.913	0.912	0.924					

Note: *, **, *** means the coefficients are significant at the levels of 0.1,0.05,0.01, respectively, with parentheses are the corresponding t-value.

Furthermore, comparing the regression results at different quantiles in the panel quantile regression reveals that as the degree of digitalization deepens, the impact on the equalization of basic public services becomes increasingly significant. However, when the equalization level of basic public services reaches the 75th percentile, the growth rate of the promoting effect of digitalization slows down, decreasing from 45.13% at the 75th percentile to 23.78%. This suggests that while digitalization is a powerful tool for achieving the equalization of basic public services and can effectively promote it, especially when the structure of digitalization and the specific elements of basic public services required by residents are reasonably and highly integrated, the marginal utility of digitalization’s promoting effect will gradually diminish once it exceeds a certain threshold.

From the perspective of control variables, increased government intervention can also promote the equalization of basic public services, but its impact is far less significant compared to the level of digital development.

### 4.3 Endogeneity test

To alleviate the endogeneity problem, when conducting regression in this paper, we have controlled as many factors affecting the equalization of basic public services as possible, as well as unobservable time effects and regional effects. However, there are many factors that affect the equalization of basic public services, and there may still be omitted variable problems. At the same time, digitalization will have an impact on the equalization of basic public services. Conversely, in the development process of the equalization of basic public services, it will further promote digital development, that is, there may be a reverse causality problem. To solve the above possible endogeneity problems, referring to the research of (Bai and Zhang, 2021) [21], this paper uses topographic relief to construct an instrumental variable for digital development. There are two considerations for choosing the interaction of topographic relief and telecommunications business volume. On the one hand, in areas with high topographic relief, infrastructure construction is more difficult and costly, and there are greater restrictions on the expansion of digital business. Digital development is more lagging compared to areas with flat terrain, which can meet the correlation requirements of instrumental variables. On the other hand, the formation of topographic relief is the result of the long-term action of natural factors and is not directly affected by endogenous factors related to digital development such as artificial digital development policies and enterprise digital strategies, which meets the exogeneity requirements of instrumental variables. Since topographic relief is cross-sectional data, referring to the practice of (Nunn and Qian, 2014) [22], this paper introduces a time-varying variable to construct a panel instrumental variable. The interaction of each province’s topographic relief and total telecommunications business volume is used as the instrumental variable IV-TRI*CT for regression. The results are shown in columns (1) and (2) of Table 6. The total telecommunications business volume is an intuitive manifestation of the level of digital development. Combining topographic relief with the total telecommunications business volume can comprehensively consider natural geographical conditions and the actual situation of digital business development, which conforms to the characteristics of correlation, exogeneity, and exclusivity of instrumental variables. Second, referring to the research method of (Peng et al., 2024; Li and Zhang, 2022) [23, 24], taking the lagged one period of digital development level as the instrumental variable IV-Digitalit-1. The results are shown in columns (3) and (4) of Table 6.

**Table 6:** Endogeneity test results.

variables	First stage (Digitalit)	Second stage (Equalit)	First stage (Digitalit)	Second stage (Equalit)
	(1)	(2)	(3)	(4)
IV-TRI*CT	0.099*** (5.424)			
**IV-Digitalit-1**			0.638*** (8.111)	
**Digitalit**		0.269*** (4.312)		0.168*** (3.092)
**Control variables**	Yes	Yes	Yes	Yes
**Year FE**	Yes	Yes	Yes	Yes
**Provincial FE**	Yes	Yes	Yes	Yes
**cons**	0.260*** (3.477)	0.332*** (7.392)	0.206*** (4.113)	0.364*** (7.046)
**Kleibergen-Paap rk LM statistic**	5.156**		26.773***	
**Kleibergen-Paap Wald rk F statistic**	29.423 (16.38)		65.782 (16.38)	
**N**	330	330	300	300
**R2**	0.963		0.968	

Note: *, **, *** means the coefficients are significant at the levels of 0.1,0.05,0.01, respectively, with parentheses are the corresponding t-value.

According to the estimation results of instrumental variables, the two-stage regression results of the two instrumental variables are both significantly positive. The P-values of Kleibergen-Paap rk LM statistic are all less than 0.05, significantly rejecting the null hypothesis of insufficient identification of instrumental variables. Moreover, the Kleibergen-Paap Wald rk F statistics are 29.423 and 65.782 respectively, which are greater than the critical value of 16.38 at the 10% level of Stock-Yogo, rejecting the null hypothesis of weak instrumental variables. This indicates that the instrumental variables selected in this paper are reasonable. After using instrumental variables to solve the endogeneity problem, digitalization can still promote the equalization of basic public services.

### 4.4 Heterogeneity test

#### 4.4.1 Regional heterogeneity analysis

Due to significant differences in economic development levels across different regions, the conditions for digital development also vary greatly, including factors such as policy support, resource endowments, and infrastructure construction. Therefore, there are considerable differences in the levels of digital development, which may lead to significant heterogeneity in their promotion of basic public services. Based on this, this paper divides the sample into four regions—eastern, central, western, and northeastern—according to the regional classification standards of the National Bureau of Statistics of China, and conducts grouped regressions. Given the large fluctuations in the level of digital development in 2021 due to the impact of the pandemic, to eliminate the influence of outliers on the regression results for different regions, the data were winsorized before regression. The regression results are shown in Table 7.

**Table 7:** Regression results were tested for regional heterogeneity.

variables	Eastern regions	Central regions	Western regions	Northeast regions
**Digitalit**	0.056* (1.894)	0.228** (2.292)	0.253** (2.150)	1.292*** (4.759)
**Constant**	0.286*** (3.775)	0.175*** (7.535)	0.154*** (2.818)	-0.662** (-2.342)
**Control variables**	Yes	Yes	Yes	Yes
**Year FE**	Yes	Yes	Yes	Yes
**Provincial FE**	Yes	Yes	Yes	Yes
**N**	110	66	121	33
**R2**	0.932	0.978	0.903	0.957

Note: *, **, *** means the coefficients are significant at the levels of 0.1,0.05,0.01, respectively, with parentheses are the corresponding t-value.

From the regression results, it is evident that digital development significantly promotes the equalization of basic public services in the eastern, central, western, and northeastern regions. In terms of heterogeneity, the impact of digital development increases sequentially from the east to the northeast, central, and western regions. The contribution of digital development to the central and western regions, as well as the northeastern region, is greater than that to the eastern region. This indicates that in less developed regions, digital development plays a more significant role in promoting the equalization of basic public services.

The reason for this result is that the original level of digitalization in the central and western regions, as well as the northeastern region, was relatively low. As digitalization penetrates deeper into these areas, it rapidly enhances the level of public service equalization by carrying the essential elements of basic public services. This is reflected in the significant promotion of public service equalization in the central and western regions and the northeastern region during this period. This further confirms that digitalization is an effective tool for achieving public service equalization. As the level of digitalization increases, it will strongly promote the improvement of public service equalization. However, it also reflects that digitalization is merely a tool and a scenario for the realization of public service elements. The improvement of public service equalization depends on the reasonable structure and high integration of digitalization and public service elements.

#### 4.4.2 Spatial complexity analysis

**Fig 1:**
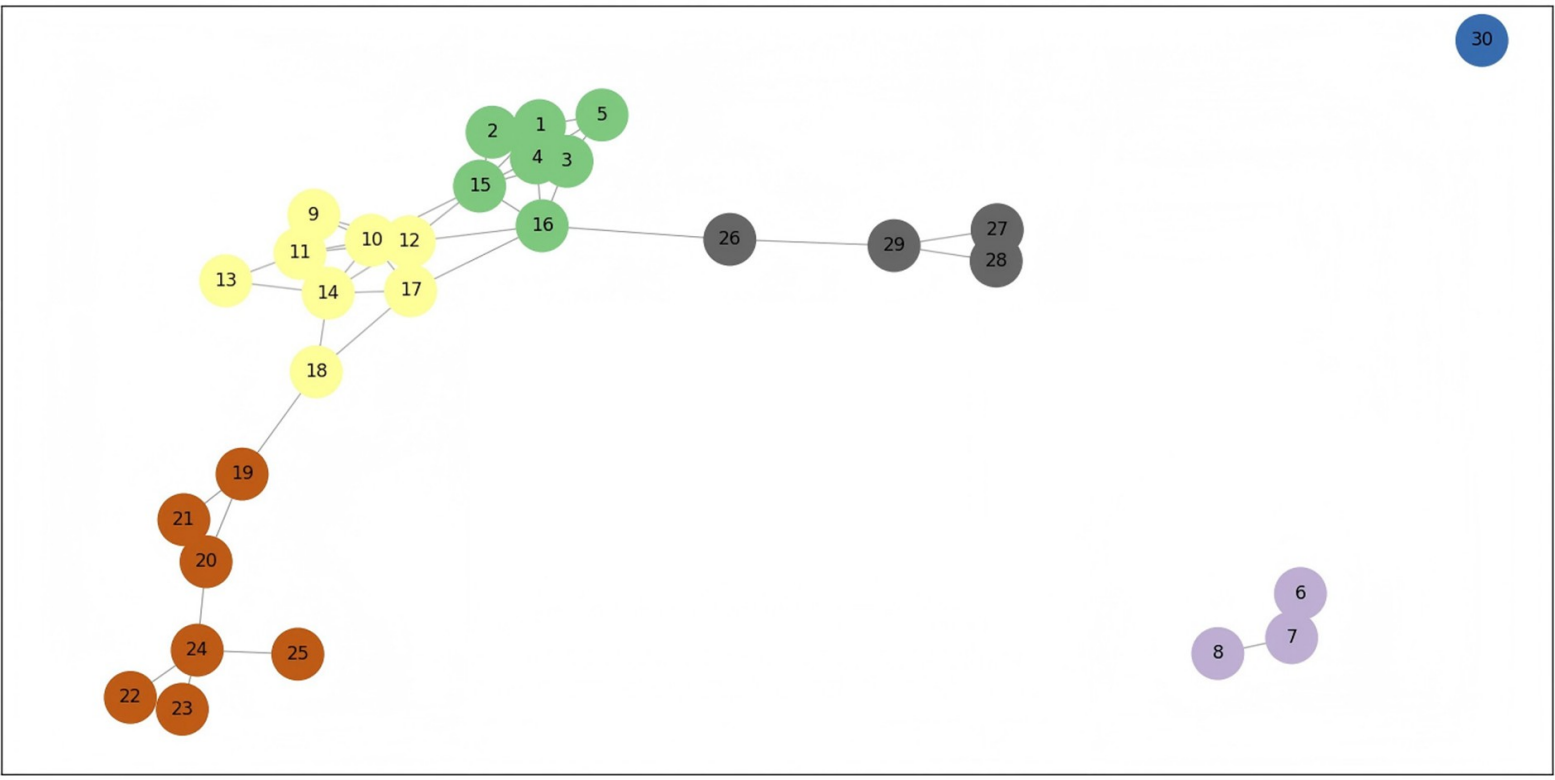
Social spatial network diagram of the impact of digitalization on the equalization of basic public services. **Note:** In the figure, the numbers 1, 2, 3, 4, 5, 6, 7, 8, 9, 10, 11, 12, 13, 14, 15, 16, 17, 18, 19, 20, 21, 22, 23, 24, 25, 26, 27, 28, 29, and 30 represent 30 provinces including Beijing, Tianjin, Hebei, Shanxi, Inner Mongolia, Liaoning, Jilin, Heilongjiang, Shanghai, Jiangsu, Zhejiang, Anhui, Fujian, Jiangxi, Shandong, Henan, Hubei, Hunan, Guangdong, Guangxi, Hainan, Chongqing, Sichuan, Guizhou, Yunnan, Shaanxi, Gansu, Qinghai, Ningxia, and Xinjiang. In the figure, geographical factors and socio-economic attribute factors are considered. Their interaction jointly determines the community structure and network connections.

**Fig 2:**
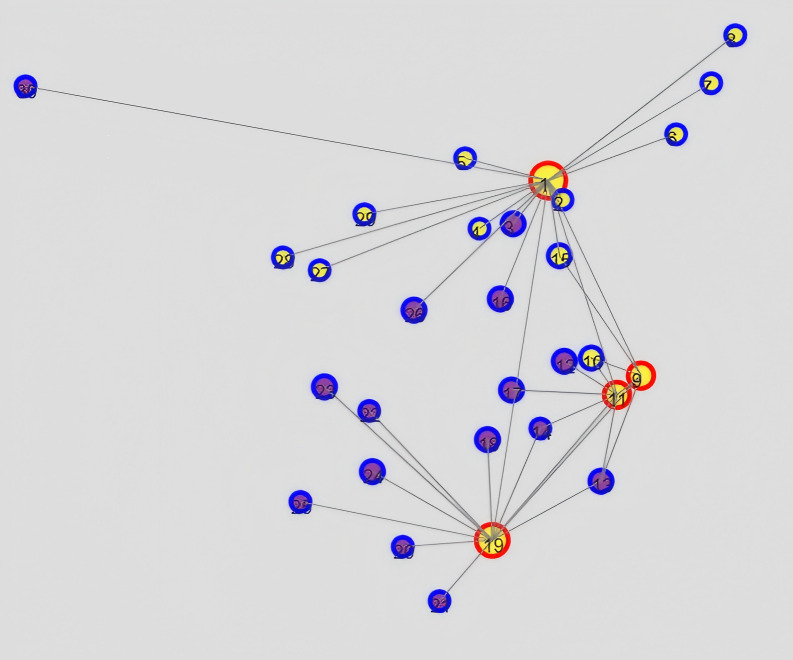
Time distortion map of the impact of digitalization on the equalization of basic public services. **Note:** In the figure, the numbers 1, 2, 3, 4, 5, 6, 7, 8, 9, 10, 11, 12, 13, 14, 15, 16, 17, 18, 19, 20, 21, 22, 23, 24, 25, 26, 27, 28, 29, and 30 represent 30 provinces including Beijing, Tianjin, Hebei, Shanxi, Inner Mongolia, Liaoning, Jilin, Heilongjiang, Shanghai, Jiangsu, Zhejiang, Anhui, Fujian, Jiangxi, Shandong, Henan, Hubei, Hunan, Guangdong, Guangxi, Hainan, Chongqing, Sichuan, Guizhou, Yunnan, Shaanxi, Gansu, Qinghai, Ningxia, and Xinjiang. Red represents regions with a comprehensive digital development level above 0.3. Blue represents regions with a comprehensive digital development level below 0.3. Yellow represents regions with an equalization level of basic public services above 0.35. Purple represents regions with an equalization level of basic public services below 0.35.

As discussed in the previous analysis, digitalization is a tool for the equalization of basic public services and is particularly crucial in the development of basic public services in the central and western regions. However, the implementation of digitalization faces certain challenges influenced by factors such as geographical location, residents’ income, and population density. This paper draws on the methods discussed by Morales et al. (2022) and Pentland et al. (2019) to analyze social spatial complexity, time-varying accessibility, and territorial cohesion [25, 26]. We construct a spatial network using information on the development level of basic public services equalization, digitalization development level, terrain ruggedness, per capita disposable income, population density, and the longitude and latitude of regions. Through community structure analysis, nodes are divided into different communities, and a social spatial network diagram is drawn, as shown in Fig 1. Additionally, considering factors such as terrain ruggedness, per capita disposable income, and population density, we select the node with the highest comprehensive digitalization score as the central node. Based on the levels of digitalization development and basic public services equalization, we calculate node displacement, travel time, and distorted distance to draw a time-distorted map reflecting the changes in the impact of digitalization on basic public services, as shown in Fig 2.

Fig 1 reveals the differences in digitalization and the equalization of basic public services across regions, as well as the complex social spatial patterns shaped by the interplay of geographical and economic factors. First, in geographical space, regions that are geographically close and have similar internal differences in digitalization and the equalization of basic public services tend to form regional clusters. However, the development of digitalization and the equalization of basic public services is constrained by territorial cohesion, leading to isolation between regions with different development levels. For example, Beijing, Tianjin, Hebei, Shanxi, Inner Mongolia, Shandong, and Henan form one regional cluster, while Shaanxi, Gansu, Qinghai, and Ningxia form another. These two clusters are connected through Henan and Shaanxi, but there is no interaction within other provinces in the same region. This isolation hinders the flow and sharing of resources and services between regions with different development levels, increasing the cost of digital infrastructure construction and exacerbating inequalities in basic public services due to income disparities among residents.

Second, there are fewer connections between different regions, especially between high-level and low-level regions. For instance, the connections between brown, yellow, green, and gray regions are single-line connections, highlighting the “digital divide” between developed and underdeveloped regions and the “low-lying areas” in basic public services. This underscores the difficulty of achieving the equalization of basic public services through digitalization across regions.

Furthermore, there are differences among provinces in terms of geographical area and population distribution, which pose varying degrees of difficulty in the coverage of digital infrastructure and the provision of basic public services. For example, in Xinjiang, where the population density is low, it is easy to encounter “island” phenomena in the advancement of digitalization for the equalization of basic public services.

The results in Fig 2 show that regions with a high comprehensive level of digital development (red nodes) have a high degree of integration with regions with a relatively high level of equalization of basic public services (yellow nodes), indicating that regions with a high level of digital development perform well in promoting the equalization of basic public services. Areas with high levels of both equalization of basic public services and digital development are closely connected. Areas with low levels in both aspects (overlapping nodes of blue and purple) are relatively dispersed and are relatively far from high-level areas. That is, in terms of time-varying accessibility, areas with high levels of digitalization and basic public services are more likely to be connected and communicate with each other. In contrast, regions with relatively low levels such as Xinjiang, Gansu, Qinghai, Ningxia, and Yunnan are mostly rugged mountainous areas, where the construction of digital infrastructure is relatively difficult, and the accessibility of realizing the equalization of basic public services through digitalization is poor. At the same time, there is an obvious differentiation in geographical space between areas with a high level of equalization of basic public services (red and yellow nodes) and areas with a low level (blue and purple nodes). This regional differentiation and unbalanced connection hinder the sharing of digital basic public service resources among regions. Low-level areas find it difficult to obtain resources and services from high-level areas, highlighting the difficulty of realizing the equalization of public services across regions through digitalization.

### 4.5 Further discussion

The regression analysis results presented earlier indicate that digitalization has a significant positive impact on the equalization of basic public services. However, does digitalization have a significant positive impact on different sectors of basic public services? To address this question, this section discusses the effects of digitalization on four secondary indicators of the equalization of basic public services. The regression results are shown in Table 8.

**Table 8:** The return result of the level of digital development to various fields.

variables	Bls_it_	Edu_it_	Ms_it_	Es_it_
**Digitalit**	0.300*** (3.037)	0.136*** (2.713)	-0.014 (-0.298)	0.159*** (3.139)
**Ed**	-0.652*** (-6.340)	0.112** (2.143)	-0.079 (-1.620)	-0.146*** (-2.761)
**Gov**	0.059 (0.762)	0.011 (0.276)	0.362*** (9.834)	-0.026 (-0.653)
**Urban**	0.850*** (6.301)	-0.144** (-2.111)	-0.244*** (-3.824)	-0.214*** (-3.094)
**str**	-0.085 (-1.553)	-0.003 (-0.113)	0.031 (1.209)	0.001 (0.053)
**Constant**	0.028 (0.495)	0.357*** (12.290)	0.200*** (7.377)	0.294*** (10.008)
**Year FE**	Yes	Yes	Yes	Yes
**Provincial FE**	Yes	Yes	Yes	Yes
**N**	330	330	330	330
**R2**	0.810	0.359	0.956	0.801

Note: *, **, *** means the coefficients are significant at the levels of 0.1,0.05,0.01, respectively, with parentheses are the corresponding t-value.

From the results, it is evident that digitalization has a significantly positive impact on basic living services, educational services, and environmental protection services at the 1% significance level, and this impact is even stronger compared to the overall regression results for basic public services presented earlier. It is also observed that the impact on basic living services is the most significant, further indicating that the market demand for basic living services is diverse and extensive, with a broad coverage area and relatively low technical barriers, making it easier for digital technologies to penetrate.

In contrast, the impact on medical services is negative and not significant. This is due to the unique characteristics of medical resources and services, which are constrained by geographical factors. Even with the full realization of digital healthcare development, people in rural and remote areas, as well as those with low incomes, may still be unable to access these services. Special medications, physical therapy, and treatment for complex diseases still require hospital visits and cannot be fully achieved through digital means, thus hindering the equitable and universal access to these services.

In order to further explore the effects of digital development on various fields and the reasons for the above results, panel quantile regression was used to perform regression for each field, and the results are shown in Table 9 below:

**Table 9:** The regression results of the digital development level on the different quantiles of each field.

variables	τ = 0.1	τ = 0.2	τ = 0.25	τ = 0.4	τ = 0.5	τ = 0.6	τ = 0.75	τ = 0.8	τ = 0.9
**Bls**_**it**_	0.148 (1.644)	0.298*** (2.699)	0.209** (2.014)	0.281** (2.417)	0.317** (2.356)	0.311** (2.439)	0.396*** (3.834)	0.381*** (3.971)	0.366** (2.439)
**Edu**_**it**_	0.108*** (3.595)	0.132*** (3.233)	0.118*** (2.800)	0.135* (1.707)	0.139* (1.667)	0.127* (1.955)	0.106* (1.699)	0.149** (2.420)	0.152*** (5.362)
**Ms**_**it**_	-0.087*** (-2.782)	-0.088* (-1.853)	-0.071 (-1.595)	-0.046 (-0.743)	-0.025 (-0.324)	0.014 (0.203)	-0.017 (-0.292)	-0.021 (-0.398)	-0.000 (-0.001)
**Es**_**it**_	0.088*** (3.551)	0.061* (1.828)	0.074** (2.270)	0.139** (2.188)	0.138 (1.564)	0.122* (1.731)	0.130*** (2.594)	0.059 (1.140)	0.160*** (6.395)
**Control variables**	Yes	Yes	Yes	Yes	Yes	Yes	Yes	Yes	Yes
**Year FE**	Yes	Yes	Yes	Yes	Yes	Yes	Yes	Yes	Yes
**Provincial FE**	Yes	Yes	Yes	Yes	Yes	Yes	Yes	Yes	Yes
**N**	330	330	330	330	330	330	330	330	330

Note: *, **, *** means the coefficients are significant at the levels of 0.1,0.05,0.01, respectively, with parentheses are the corresponding t-value.

From the results of quantile regression, it is observed that the impact on healthcare services remains negative and significant only at the 0.1 and 0.2 quantiles, consistent with the benchmark regression results mentioned earlier. The impact of digitalization on basic living services, educational services, healthcare services, and environmental protection services shows significant differences across different quantiles. The role of digitalization in promoting the equalization of educational and environmental protection services generally exhibits a fluctuating upward trend. The influence of digitalization on the equalization of residents’ basic living services shows a pattern of first increasing and then decreasing, following a law of diminishing marginal utility.

For educational and environmental protection services, an increase in the degree of digitalization helps to improve the level of equalization of these services, with their promotion showing a general trend of fluctuating upward development. This indicates that the deep application of digital technology in the field of education and its integration with educational resources have effectively broken down restrictions related to geography, economy, and resources. Through the internet, students can easily access rich online educational resources, ensuring that students in both urban and rural areas can obtain equal learning opportunities and resources through digital educational services, thus achieving educational equality. Similarly, the application of digital technology in the environmental protection sector not only enables comprehensive environmental monitoring but also makes waste management and sewage treatment more convenient and efficient in both urban and rural areas, breaking down geographical barriers. Additionally, smart irrigation systems and vegetation health monitoring contribute to maintaining green coverage and green space in developed areas, ensuring that every citizen enjoys fresher air quality and a cleaner living environment.

For residents’ basic living services, the impact of digitalization shows a fluctuating development trend of first rising and then declining. At the 0.75 quantile, the promoting effect of digitalization on basic living services reaches its maximum, after which it begins to decrease. This reflects the process where, as a tool for achieving the equalization of basic public services, digitalization effectively carries relevant elements of basic public services to achieve their popularization. When the degree of digitalization is low, its utility as an element for achieving the equalization of basic public services is significant, leading to a rapid rise in the equalization of basic public services. However, when digitalization reaches a certain level, the law of diminishing marginal utility takes effect, causing the equalization of basic public services to gradually decline from a rapid increase, indicating that the role of digitalization in the equalization of basic public services has reached a critical point. This is the manifestation of the law of diminishing marginal utility of digitalization in the field of residents’ basic living services.

The impact of digitalization on the equalization of basic public healthcare services is negatively significant at the 0.1 and 0.2 quantiles, while it is insignificant at other quantiles. First, the introduction and improvement of digital technology allow more medical services to be provided online, reducing reliance on physical medical institutions. The multifunctional applications of digital hospitals mean that fewer hospital facilities are needed in a region; instead, one or two highly digitalized hospitals can adequately support the medical needs of the entire area. Second, the widespread availability of digital medical services enhances accessibility to healthcare, allowing people to receive remote medical services without leaving their homes, and many medical needs can now be met within families or communities, leading to a reduction in hospital bed numbers. Third, the development of digitalization has effectively disseminated medical and health knowledge, reducing the incidence of common diseases. Therefore, the suppression of medical resources such as hospitals and hospital beds by digitalization actually indicates a further optimization of medical resource allocation and utilization, enhancing the accessibility of medical services and promoting the equalization of medical care.

## 5. Research conclusions and enlightenment

### 5.1 Research conclusion

As the level of digitalization continues to improve, promoting the deep integration of digital technology with basic public services and facilitating the widespread application of digital technology in the field of basic public services is an important approach to achieving equalized development of basic public services. It is also a key focus for achieving stable and sustainable high-quality economic and social development. This paper, based on provincial panel data from China’s basic public services and digitalization development from 2013 to 2023, uses multiple models to empirically test the relationship between the level of digitalization and the equalized development of basic public services, as well as the mechanism by which digitalization affects the equalization of basic public services.

The research findings indicate that, first, digitalization can significantly promote the process of equalizing basic public services. This conclusion remains valid after endogeneity testing and robustness testing, and the results of panel quantile regression show that within a certain range, as the degree of digitalization increases, its promoting effect on the equalization of basic public services also becomes stronger. However, digitalization is merely a tool for promoting the equalization of public services, and its promoting effect exhibits diminishing marginal utility. Second, regarding regional heterogeneity, the impact of digitalization on the east, northeast, central, and western regions increases sequentially, and the contribution of digitalization to the central and western regions as well as the northeast is greater than that to the eastern region. That is, the more backward and underdeveloped a region is, the greater the role of digitalization in promoting the equalization of basic public services, providing policy basis for the western region and economically underdeveloped areas to advance the equalization of basic public services through increased digitalization.

Third, regions that are geographically close and have similar internal differences in the development of digitalization and the equalization of basic public services tend to form regional cohesion. However, the development of digitalization and the equalization of basic public services are constrained by territorial cohesion, leading to isolation between regions at different levels of development. This manifests as a “digital divide” between developed and underdeveloped regions and a “low-lying area” phenomenon in basic public services. Moreover, regions with both high levels of digitalization and equalization of basic public services exhibit strong time-varying accessibility, while low-level regions are scattered and relatively far from high-level regions, making it difficult to achieve equalization of basic public services through digitalization. Fourth, in terms of the heterogeneity of the effects of digitalization on different sectors of basic public services, due to the characteristics of large market demand, wide coverage, and low technical barriers in basic living services, digital technology can easily penetrate, resulting in the greatest promoting effect. This is followed by environmental protection services and educational services. Due to geographical constraints, medical resources, and the particularity of medical services, the effect on medical services is not significant.

Additionally, quantile regression shows that the promoting effect of digitalization on the equalization of educational and environmental protection services is generally increasing with fluctuations, while the promoting effect on the equalization of basic living services follows a law of diminishing marginal utility. Although digitalization appears to inhibit indicators of medical service equalization such as the number of beds and doctors, this actually reflects the deepening of residents’ use of digital applications, which has improved the accessibility of medical services, allowing more people to enjoy better medical resources through the internet and other means, thereby enhancing the level of medical service equalization.

### 5.2 Policy recommendations

Digital development has become a crucial force driving social progress and economic growth. Not only has digital development transformed people’s lifestyles and work methods, but it has also had a profound impact on the provision and equalization of basic public services. As digital technologies are increasingly applied across various fields, the benefits of the digital transformation of the economy and society are becoming more abundant. The domain of basic public services should also keep pace with the times and accelerate its digital transformation.

a)Accelerate the construction of digital infrastructure. The development of digital infrastructure is the foundation for overcoming the digital divide. Only with well-developed digital infrastructure can the equalization of basic public services be achieved. The government and all sectors should increase investment in the construction of digital infrastructure, particularly in remote and rural areas where digital development lags behind. Efforts should be made to expedite the deployment of 5G networks, big data, cloud computing, and other infrastructure, as well as the innovative application of smart public service devices, to ensure that everyone can benefit from the convenience and opportunities brought by digital development. Additionally, there should be a greater emphasis on promoting digital literacy education, providing necessary training to help people master digital technologies and fully utilize the resources provided by digital development.

b) Promote the application of digital technologies in basic public services. By leveraging digital technologies, it is possible to break down geographical barriers and enable the broader application of high-quality public service resources, thus promoting shared prosperity. For example, establishing a digital healthcare system can provide online medical consultations and telemedicine services, ensuring that residents in remote areas can access quality healthcare resources. Furthermore, by promoting the widespread application of digital education, online educational resources and remote teaching can be provided to ensure that every student has access to good educational opportunities, especially in remote and impoverished areas, where everyone should have the right to receive an equal education.

c) The government should strengthen supervision and promote the standardized use of digital technologies in the public service sector. While digital development promotes the equalization of public services and supports the realization of shared prosperity, it also brings new challenges and risks, such as data security and privacy protection. The government should enhance coordination among departments, accelerate the establishment of data sharing mechanisms, and improve relevant laws, regulations, and regulatory frameworks to ensure that digital development plays a positive role in protecting citizens’ rights and maintaining social stability. Additionally, the government should strengthen oversight of digital service providers and their staff to prevent the commercialization of digital public services, which could exacerbate inequalities among the public and in society. It is essential to ensure the correct application of digital technologies in the basic public service sector, providing safe, reliable, and inclusive basic public services.

## 6. Research limitations and future prospects

This study empirically examines the empowering role of digital development in the process of achieving equalization of basic public services and its impact effects in different fields, providing valuable insights for related research. However, there are still several limitations. For instance, due to data availability, this study only collected provincial-level sample data, resulting in a limited sample size and a restricted range of dimensions involved in basic public services. Future research will evaluate samples at the city and county levels in China, and conduct a detailed analysis of regional differences and dynamic changes. Additionally, it will further expand the measurement dimensions of basic public services, incorporating aspects such as labor employment and entrepreneurship, social insurance services, and housing security for a more comprehensive study. This research will provide more targeted policy recommendations to mitigate the impact of the digital divide and promote the equalization of basic public services.
